# A germline mutation in Rab43 gene identified from a cancer family predisposes to a hereditary liver-colon cancer syndrome

**DOI:** 10.1186/s12885-019-5845-4

**Published:** 2019-06-21

**Authors:** Yanting Jiang, Yue Sun, Jiandong Hu, Nan Yu, Hui Liu, Jiankun Fan, Xuelian Ning, Yilan Li, Baogang Liu, Yihua Sun, Jinwei Zhang, Xiaohong Qiu, Songbin Fu, Chunshui Zhou, Hui Xu

**Affiliations:** 1 The Laboratory of Medical Genetics, Harbin Medical University, Harbin, 150081 China; 2 The 2th Affiliated Hospital, Harbin Medical University, Harbin, 150001 China; 3 The Tumor Hospital, Harbin Medical University, Harbin, 150081 China; 4 Key laboratory of preservation of human genetic resources and disease control in China, Harbin Medical University, Ministry of Education, Harbin, 150081 China

**Keywords:** Hereditary cancer syndrome, Germline mutation, Whole-exome sequencing, Rab43

## Abstract

**Background:**

Hereditary cancer syndromes have inherited germline mutations which predispose to benign and malignant tumors. Understanding of the molecular causes in hereditary cancer syndromes has advanced cancer treatment and prevention. However, the causal genes of many hereditary cancer syndromes remain unknown due to their rare frequency of mutation.

**Methods:**

A large Chinese family with a history of hereditary liver-colon cancer syndrome was studied. The genomic DNA was extracted from the blood samples of involved family members, whole-exome sequencing was performed to identify genetic variants. Functional validation of a candidate variant was carried out using gene expression, gene knockout and immunohistochemistry.

**Results:**

The whole-exome of the proband diagnosed with colon cancer was sequenced in comparison with his mother. A total of 13 SNVs and 16 InDels were identified. Among these variants, we focused on a mutation of Rab43 gene, a GTPase family member involving in protein trafficking, for further validation. Sanger DNA sequencing confirmed a mutation (c: 128810106C > T, p: A158T) occurred in one allele of Rab43 gene from the proband, that heterozygous mutation also was verified in the genome of the proband’s deceased father with liver cancer, but not in his healthy mother and sister. Ectopic expression of the Rab43 A158T mutant in Huh7 cells led to more enhanced cell growth, proliferation and migration compared to the expression of wild type Rab43. Conversely, knockout of Rab43 in HepG2 cells resulted in slow cell growth and multiple nuclei formation and impaired activation of Akt. Finally, a positive correlation between the expression levels of Rab43 protein and cancer development in that family was confirmed.

**Conclusions:**

A germline mutation of Rab43 gene is identified to be associated with the onset of a familial liver-colon cancer syndrome. Our finding points to a potential role of protein trafficking in the tumorigenesis of the familial cancer syndrome, and helps the genetic counseling to the affected family members.

**Electronic supplementary material:**

The online version of this article (10.1186/s12885-019-5845-4) contains supplementary material, which is available to authorized users.

## Background

Hereditary cancer syndromes refer to a group of cancer patients harboring germline mutations and these mutations predispose carriers to several benign and malignant tumors. So far, only a small number of hereditary cancer syndromes are extensively studied. For example, mutations in the TP53 gene are the cause of about 70% of Li-Fraumeni cancer syndrome [1]. Mutations in BRCA1 (17q21) and BRCA2 (13q12.3) have been linked to hereditary breast and ovarian cancer syndrome [2, 3]. Familial Adenomatous Polyposis (FAP) is caused by mutations in the adenomatous polyposis coli (APC) gene [4]. Germline mutations in DNA mismatch repair genes including MLH1, MSH2*,* MSH6*,* or PMS2 give rise to Lynch syndrome [5]. The understanding of molecular aberrations in hereditary cancer syndromes has dramatically advanced targeted cancer therapies in recent years. The knowledge gained from genetics of hereditary cancer syndromes can also help genetic counseling and cancer prevention [6]. However, despite a few of well-defined hereditary cancer syndromes described above, the causal mutations of the majority hereditary cancer syndromes are largely unclear due to their extremely low frequency of mutation. Here, we identified a large Chinese cancer family with a clear hereditary family history of liver and colon cancer. We employed whole-exome sequencing technology to analyze the genomic DNA from the blood of the proband in comparison with his mother and discovered a heterozygous germline mutation of Rab43 gene is associated with the onset of this familial liver and colon cancer syndrome.

## Methods

### Patients and DNA samples

The family with a history of hereditary cancers consisted of two persons with colorectal cancer and four persons with liver cancer spanning in three consecutive generations. Blood samples were collected from available family members except the fourth generation members because of their young ages and other concerns. For the proband’s deceased father diagnosed with liver cancer, DNA and liver tissue were obtained from the pathological archives of a local hospital where the patient was treated. All living participants involved in this research signed written informed consent forms. This research was approved by the Harbin Medical University ethics committee in accordance with Helsinki Declaration.

### Genomic DNA preparation and whole-exome sequencing

Genomic DNA was extracted from peripheral leukocytes by DNA purification Kit (Qiagen) according to the manufactrue’s instructions. DNA concentrations, DNA purity and integrity were assessed by spectrophotometry (Nanodrop) and agarose gel electrophoresis and met the DNA sequencing requirements. The gDNA library was prepared using Ion Xpress Plus Fragment Library Kit (Thermo Fisher Scientific). Exome enrichment was performed by SureSelect™ target enrichment system (Illumina) in accordance with the manufacturer’s instructions. The enriched DNA samples were sequenced via paired 126 bp sequencing using a Hiseq2500 Sequencing System (Illumina).

### Whole-exome sequencing data filtering analysis

A binary sequence alignment map (BAM) file was generated by comparison of the paired DNA samples. Somatic SNVs and indels were detected by comparison of the paired BAM files. The sequence reads were aligned to the human reference genome (UCSChg19) using the Burrows-Wheeler Aligner (BWA) with default parameters. Variants were identified using the Genome Analysis Toolkit (GATK). SNVs and indels were analyzed using Haplotype Caller and data were filtered by variant quality score recalibration. Relevant mutations in all of the genes were subsequently prioritized manually.

### Variant validation, allele segregation analysis

Exome sequencing results for prioritized variants were validated using specific primers for amplification by polymerase chain reaction and Sanger sequencing. Segregation analysis of the prioritized variants was performed in additional family members (including a deceased member with hepatocellular cancer) whenever genomic DNA was available.

### Immunohistochemistry (IHC)

IHC was carried out on paraffin-embedded sections of colon, liver cancer and their adjacent non-cancerous tissues obtained through surgery to investigate the expression levels of endogenous Rab43 with primary antibodies against human Rab43. A total of fifteen cancerous and fifteen adjacent non-cancerous tissue sections (slides) were analyzed for each liver or colon cancer patient. Each slide was blinded read under light microscopy by two board-certified pathologists. Ten randomly picked view fields from each slide were assessed. The staining intensity and proportion of positively stained cells were evaluated as described previously with minor modifications [7]. The intensity of Rab43 staining (brown color) was scored as follows: 0, negative; 1, weak; 2, moderate; 3, strong; And the percentage of positively stained cells was scored as follows: 0, < 5%; 1, 5–25%; 2, 26–50%; 3, 51–75%; 4, > 75%. The sum of each intensity score multiplied by its corresponding positive percentage score was regarded as the Rab43 staining score for the viewed field and assigned as follows: - for (0); + for (1–2); ++ for (3–5); +++ for (6–8); ++++ for (9–12).

### Cell lines and tissue culture

The human cancer cell lines used in our research including HeLa (CCL-2), A549 (CCL-185), Skov-3 (HTB-77) and HepG2 (HB-8065) were purchased from American Type Culture Collection (ATCC, VA, USA). Cell line Huh7 (BNCC337690) was purchased from the Cell Bank of the Chinese Academy of Sciences (Shanghai, China). These cell lines were free of mycoplasma contamination as tested by a PCR method prior to our research. Cells were maintained in Dulbecco’s Modified Eagle Medium (Gibco, NY, USA) supplemented with 10% fetal bovine serum (Gibco), 100 unit/ml penicillin and 0.1% (w/v) streptomycin (Gibco) in a 5% CO_2_ humidified incubator at 37 °C.

### Plasmid construction and cell line generation

The human Crispr-Cas9 guide RNA was designed based on the coding gene of Rab43 (GenBank accession number: AY166852). The primer pair for gRNA was: Rab43-cri-F3: 5′-caccggctactaccgcagtgccaat-3′, Rab43-cri-R3: 5′- aaacattggcactgcgg tagtagcc-3′. The primer pair was annealed using the following parameters: denatured at 95 °C for 5 min and cooled down to room temperature. The annealed primer pair was cloned into the Lenti-Crispr-V2 vector (a gift from Dr. Feng Zhang of MIT, USA) through BsmI restriction sites. The gRNA cloning was verified by Sanger sequencing. HepG2 cells were transfected with the plasmid Lenti-Crispr-V2-Rab43–3 by lipofectamin-3000 according to the manufacturer’s instruction. The stable transfected cells were selected by puromycin, and the resulting cell clones with desired mutations in Rab43 gene were verified by Sanger sequencing and Western blotting.

Wild type Rab43 gene was amplified from a gift plasmid (from Dr. John Cox of University of Tennessee) containing the wild type ORF of Rab43 by using two following specific primers with XhoI and EcoRI restriction sites. Rab43-cpXhoI-F: 5′-aattctcgagatggcagggccgggccca-3′, Rab43-cpEcoRI-R: 5′-aattgaattctcagcacccgcagcc ccag-3′. Wild type Rab43 ORF was subcloned into pcDNA3.1 (−)-3 × Flag via XhoI and EcoRI sites. Site-directed mutagenesis using following primers with the desired mutations (F: 5′- ctatgacatcctgtgtaccattgagacgtctg-3′, R: 5′-cagacgtctcaatggtacacaggat gtcatag-3′) was performed for Rab43 A158T mutant generation. The Huh-7 cells transfected with pcDNA3.1-(−)-3 × Flag-Rab43 A158T were selected by G418. Following primer pair was used for generating Crispr resistance Rab43 ORF in pcDNA3.1-(−)-3 × Flag plasmid. Cri-resi-3F: 5′-agctactatagatcagctgcagccgccatccttg-3′, Cri-resi-3R: 5′-caaggatggcggctgcagctgatctatagtagct-3′.

### Colony formation assay

Cells were seeded at a density of 500 cells/well in 6-well plates. After 2 weeks, the cells were fixed with 4 ml of 4% paraformaldehyde for 15 min, and stained with 0.2% crystal violet for 10 min. The numbers of visible colonies with more than 50 cells per colony were counted. Colony formation was calculated from three independent experiments.

### Cell proliferation assay

Cells were seeded in 96-well plates at the density of 1000 cells/well and incubated for the next day. The cell proliferation assays (MTT) were performed on day 1, 3 and 5 after seeding. Experiments were carried out in triplicate.

### In vitro cell migration assay

Cell migration assay was performed by using wound healing method as described previously [8]. Briefly, cells were seeded into a six-well plate at a density of 5 × 10^5^/well and allowed to culture overnight. Cells were then treated with mitomycin C (final concentration at 50μg/ml, Sigma) to inhibit cell proliferation for 2 h before scratches were generated by applying a 200 μL pipette tip. Cells were washed twice with PBS buffer in order to remove all the dislodged cells. Photographs were taken at 0 h, 24 h and 48 h after wounding. Migration of the cells was measured by determining the wound healing areas of control cells versus Rab43 overexpressed Huh7 cells. Assays were performed in triplicate.

### Western blotting

Cells were lysed in RIPA lysis buffer. The lysates were sonicated and centrifuged at 12,000 rpm and 4 °C for 10 min. Cell lysates were quantified using BCA protein assay kit (Pierce) and normalized for protein content by RIPA buffer. After separation on SDS-PAGE, the proteins were then transferred to PVDF membrane (Millipore). The membranes were incubated with the primary antibody (1:1000) probing Rab43 (Santa Cruz), Akt (Cell Signaling), p-Akt (Ser473) (Cell Signaling),, phos-pP70S6K (Cell Signaling) or GAPDH (Sigma) over night at 4 °C and incubated with the fluorescein conjugated secondary antibodies (1:5000) for 1 h at room temperature. The proteins were detected and photographed using Odyssey imaging system (Li-COR biosciences). GAPDH was used as loading control. The signal intensity was quantified using the Image J software (NIH).

### Statistical analysis

For statistical evaluation, data are expressed as mean ± standard deviation (SD). Statistical differences for comparison of cell growth, colony formation and wound healing were determined by Student’s t test or one-way ANOVA test. The Rab43 expression levels in colon and liver tissues were examined by Mann–Whitney test. All analyses were performed using SPSS for Windows 17.0 software (SPSS Inc., Chicago, IL, USA). *p* < 0.05 was considered statistically significant.

## Results

### Identification of germline mutations in a Chinese hereditary cancer family

**Fig. 1:**
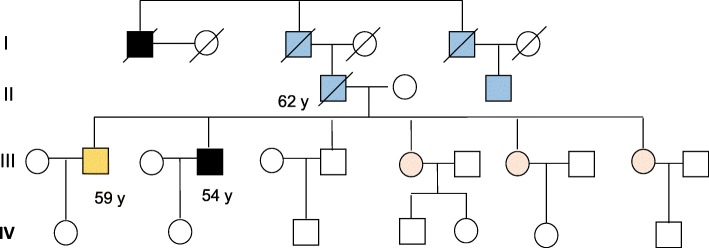
The pedigree of a large Chinese cancer family with a liver-colon cancer syndrome A clear dominant hereditary pattern of liver-colon cancer syndrome was observed in the family. Square: male; Circle: female; Blue square: diagnosed with liver cancer; Black square: diagnosed with colorectal cancer; Yellow square: diagnosed with colon adenomas; A square or a circle with a slash: deceased; The number under each affected individual indicates his/her age at diagnosis

We identified a large Chinese family with four generations living in Northeast of China with a clear history of hereditary liver and colon cancers. As of the date when blood samples were collected, a total of two colon cancers and four liver cancers and one colon adenoma have been diagnosed spanning the first three consecutive generations (Fig. 1). Those diseases display an obvious autosomal dominant pattern of inheritance. Furthermore, there is no affected member in the fourth generation most likely due to their relative young ages.

**Fig. 2:**
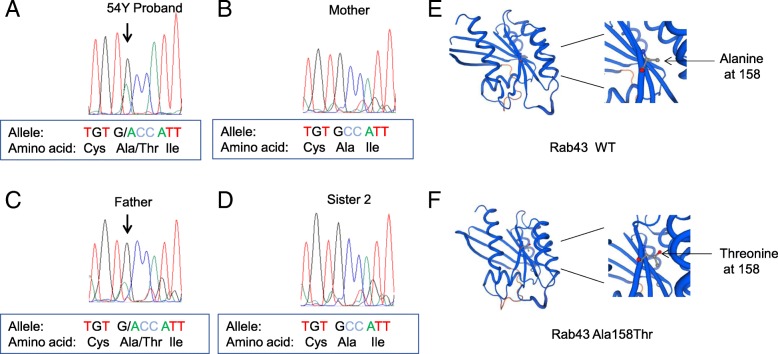
Verification of the germline mutation in Rab43 gene by Sanger sequencing and structural comparison of wild type Rab43 with the identified Rab43 mutant protein (**a-d**) The Sanger sequencing chromatograms of Rab43 gene from the proband, his parents and one sister were displayed by the ChromasPro software. The mutation site was indicated with a black arrow. The corresponding codon sequences and coding amino acids were placed under each chromatogram. Whereas the two alleles of Rab43 gene from his mother and sister are wild type, one allele of Rab43 gene from the proband and his father harbors the identified mutation. (**e-f**) Comparison of the predicted crystal structures between wild type Rab43 and Rab43 A158T mutant. The local structure surrounding residue 158 was displaced in the enlarged insets. Lines with Black arrows indicate the position of residue 158. The side chain of Alanine at 158 or Threonine at 158 was highlighted by red dots. The pictures were rendered with PyMOL (v.0.99rc6)

We sequenced the whole-exome of the proband who was diagnosed with colon cancer at the age of 54. Meanwhile, his mother’s whole-exome sequencing was performed in parallel for comparison. In addition, we also compared our sequencing results with the hg19 genome database maintained by University of California Santa Cruz as a third party reference. We used six bioinformatics tools to select for deleterious mutations, including PhyloP (score > 0.85), SIFT (score < 0.05), PolyPhen (score > 0.85), GERP (score > 2), Mutation Taster (score > 0.5), and LRT (score > 0.9), and only those mutations with four or more positive deleterious predictions were further considered. Eventually, we obtained 13 SNVs from 13 genes (Table 1) and 16 Indels from 15 genes (Table 2). Among these candidate genes, based on available information including their known functions, potential protein interactions and predicted effects on protein structure, we focused on the variant of Rab43 for further analysis. There is a C > T transition occurred on the anti-sense strand of one Rab43 allele in the genome of the proband, this mutation leads to substitution of alanine by threonine at the 158 residue on Rab43 protein. The heterozygous mutation status of Rab43 gene in the proband was confirmed by Sanger sequencing (Fig. 2a). The segregation of the mutant allele was analyzed in additional family members except all members from the fourth generation of that family. While the patient’s healthy mother and two sisters are homozygous for wild type Rab43 alleles, the genome of his father who died from liver cancer indeed harbors one of the mutant allele of Rab43 (Fig. 2b-d). These data demonstrated the mutant allele of Rab43 is clearly co-segregated with the familial cancer syndrome, though the segregation information is not available in the fourth generation currently. In addition, we performed protein structure analysis by using PyMol software [9] and found there is no overall structural change with the threonine substitution of alanine at residue 158 on Rab43 protein (Fig. 2e-f). There are many known oncogenic mutations that would interfere with the functions rather than the overall structures of the affected proteins. One prominent example of such mutations is the oncogenic Ras G12 V mutant. A previous study reported the overall structures between the GDP bound form of the normal Ras protein and the GDP bound form of the Ras G12 V are identical, suggesting it is the side chain on Valine12 rather than the conformation changes would impair the intrinsic GTPase activity associated with Ras [10]. In the case of Rab43 A158T, we propose it is the hydroxyl group on Threonine 158 rather than any structural changes that would enhance the functions associated with Rab43 protein, hinting strongly the identified Rab43 variant might be oncogenic with a gain of function.

**Table 1:** SNVs identified from the 54 year old proband in comparison with his mother

Chr	Position	rsID	Gene	DNA change	AA change
1	151,090,537	rs2864699	GABPB2	G/C	Q/H
1	248,569,476	–	OR2T1	G/T	D/Y
3	128,810,106	rs970572	RAB43	C/T ^a^	A/T
4	17,609,087	–	LAP3	C/T	H/Y
7	6,692,019	rs2250356	ZNF316	C/G	–
11	118,401,572	rs535760315	TTC36	T/C	C/R
12	110,206,393	rs824998	FAM222A	G/A	C/Y
15	102,359,116	rs1370063626	OR4F15	C/T	H/Y
16	66,776,341	rs8055189	DYNC1LI2	A/G	F/L
16	67,860,078	rs188707401	TSNAXIP1	C/T	R/C
16	87,466,766	rs7499131	ZCCHC14	T/G	K/Q
16	87,760,390	rs4843689	KLHDC4	T/C	Y/C
22	17,600,584	–	CECR6	G/T	D/E

*Chr* chromosome and its number, *rsID* the indel ID obtained from dbSNP135 database, *AA change* amino acid change

^a^The C to T transition in Rab43 gene reflects the sequencing results of the DNA changes on the anti-sense strand of Rab43 gene

**Table 2:** InDels identified from the 54 year old proband in comparisonwith his mother

Chr	Position	rsID	Gene	DNA change	AA change
3	54,959,059	rs370607786	LRTM1	T/−	H > Fs
3	75,787,349–75,787,353	–	ZNF717	CCCTG/−	T > Fs
3	97,887,844	rs377659479	OR5H15	T/−	F > Fs
4	70,360,942	–	UGT2B4	T/−	N > Fs
8	10,480,295	rs201192645	RP1L1	−/G	P > Fs
11	4,566,460	rs77893620	OR52M1	T/−	F > Fs
14	38,679,763	–	SSTR1	−/GCTCT	T > delins TLX
17	62,855,003	–	LRRC37A3	−/A	–
19	41,018,845	–	SPTBN4	C/−	Q > Fs
20	168,625	rs11396059	DEFB128	−/T	H > Fs
20	1,592,151–1,592,152	–	SIRPB1	G/−	L > Fs
20	1,592,154	–	SIRPB1	−/AC	D > Fs
20	2,674,430	–	EBF4	T/−	–
20	50,701,795	–	ZFP64	G/−	P > Fs
21	11,029,597	rs60459764	BAGE2,BAGE3	C/−	–
22	43,926,809–43,926,813	–	EFCAB6	AGAGT/−	Y > Fs

*Chr* chromosome and its number, *rsID* the indel ID obtained from dbSNP135 database, *AA change* amino acid change

### Rab43 A158T variant exhibits oncogenic activity in liver cancer cells

**Fig. 3:**
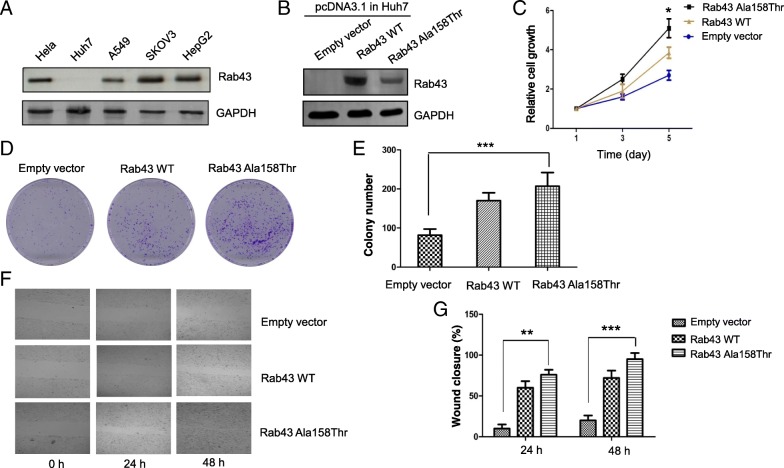
Ectopic expression of Rab43 A158T in Huh7 cells enhances cell growth, colony formation and migration (**a**) Expression levels of endogenous Rab43 in a variety of human cancer cell lines. Western blotting confirmed an extremely low level of endogenous Rab43 expression in Huh7 cells. **b** Ectopic expression of wild type Rab43 and Rab43 A158T mutant in Huh7 cells. Note: the expression level of wild type Rab43 is higher than that of Rab43 A158T mutant. **c** Huh7 cell growth was stimulated by over-expression of Rab43 A158T mutant. Cell growth was measured by MTT assay, the growth difference was significant between empty vector and Rab43 A158T mutant transformed cells on day 5 (* *p* < 0.05 by ANOVA test). **d-e** Huh7 proliferation and colony formation was increased by over-expression of Rab43 A158T mutant. Three representative colony formation plates stained with crystal blue are presented. Cells were stained on day 10 after plating, colonies with more than fifty cells per colony were counted and the average colony number from each cell line was plotted in (**e**). Data are from three independent experiments and presented as average ± SD (standard deviation), *** *p* < 0.001 indicated statistically significant by ANOVA test. **f-g** Huh7 cell migration was enhanced by over-expression of Rab43 A158T mutant. Cell migration was determined by wound healing assay. The percentages of wound closure in each cell line were measured at indicated time points post wound creation. Representative wound healing images were shown in F and average wound closure rates of each cell line at the indicated times were plotted in (**g**). Data are from three independent experiments and presented as average ± SD (standard deviation), **p < 0.01 and *** *p* < 0.001 indicated statistically significant by ANOVA test

Rab43 is a Rab family related small GTPase and regulates the sorting of a subset of protein cargo through the medial Golgi [11–13]. Similar to mutations occurred on oncogene Ras [14, 15], the Rab43 mutation identified in our study might be oncogenic. In order to determine the functional consequence of Rab43 A158T, we subcloned wild type Rab43 gene and generated Rab43 A158T mutant gene by site directed mutagenesis. We screened an array of human cancer cell lines and found the expression levels of Rab43 are varied dramatically (Fig. 3a). Ectopically expressed both wild type Rab43 and Rab43 A158T mutant proteins in liver cancer cell line Huh7 with a very low level of endogenous Rab43 protein enhanced cell growth and proliferation (Fig. 3b-c), stimulated the colony formation on cultured plates (Fig. 3d-e) and promoted the migration of the transfected Huh7 cells in vitro (Fig. 3f-g). More strikingly, compared to the effects of the expressed wild type Rab43, the expressed Rab43 A158T mutant protein tends to confer more malignant transformation capacity to the transfected Huh7 cells (Fig. 3c-g). Interestingly, overexpression of Rab43A158T in another liver cancer cell line HepG2 with a high level of endogenous Rab43 expression has minimal effects on the growth, proliferation, colony formation and migration of the transfected HepG2 cells, indicating Rab43 A158T variant can substitute the function of WT Rab43 once wild type Rab43 is insufficient or lost completely. Taken together, our results strongly support Rab43 A158T variant possesses oncogenic activity and plays a driving role in the onset of the studied cancer syndrome, consistent with the autosomal dominant pattern of inheritance observed in that family.

### Rab43 is required for Akt activation and Akt signaling

**Fig. 4:**
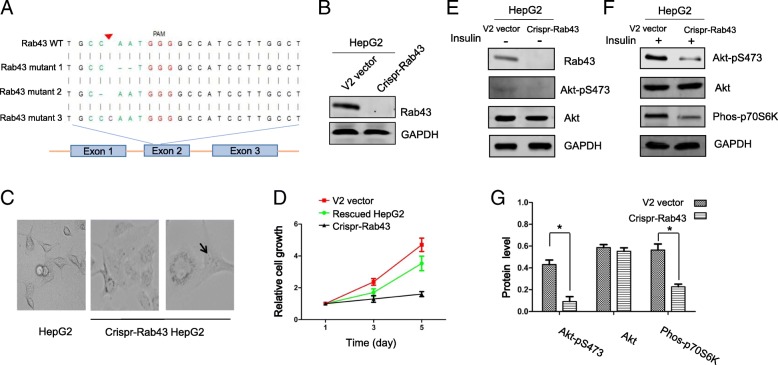
Loss of Rab43 retards cell growth and proliferation through impairment of Akt activation (**a**) Generation of Rab43 knockout HepG2 cell line by Crispr-Cas9 gene editing, a Rab43 knockout cell line was established and verified by Sanger DNA sequencing with three different reading frame shift mutations as shown in (**a**). The red arrow indicates the location of a deoxycytidine triphosphate insertion. **b** Western blot confirmed Rab43 protein is completely lost in the Crispr-Rab43 HepG2 cells. **c-d** Rab43 knockout HepG2 cells display an enlarged and flattened phenotype with slow growth, some cells exhibit multiple nuclei as indicated by a black arrow. The slow growth of Rab43 knockout cells could be reversed by over-expressing a Crispr-resistant Rab43 gene. **e-g** Activation of Akt and Akt signaling is impaired in Rab43 knockout HepG2 cells. Basal level of Akt activation was barely detectable in Crispr-Rab43 cells without insulin stimulation by Western blotting in (**e**). The indicated cells were serum-starved overnight and re-stimulated with 100 nM insulin for 30 min. The expression levels of Akt-S473, Akt and phos-p70S6K were detected by Western blot in (**f**), quantified by densitometry scanning, normalized to GAPDH signal and plotted in (**g**). Quantification data were based on three independent experiments. The activation differences of Akt and phos-p70S6K between V2 control and Crispr-Rab43 cells post insulin stimulation were statistically significant (*p < 0.05 by Student’s t test)

Rab43 has been implicated in glioma development previously [16], but the underlying mechanism is unclear. To further elucidate the molecular mechanism of Rab43 variant mediated tumorigenesis, we eliminated the Rab43 expression in HepG2 cells by Crispr-Cas9 gene editing technology targeting the exon 2 of Rab43 gene (Fig. 4a) and established a HepG2 Rab43 knockout cell line with 3 different reading frame shift mutations in the coding region of Rab43 gene, which cause complete loss of endogenous Rab43 protein expression in the Crispr-Rab43 HepG2 cells (Fig. 4a-b, for detailed Sanger sequencing results see Additional file 1: Figure S1). To our surprise, the Rab43 knockout HepG2 cells are enlarged and flattened with slow growth (Fig. 4c-d). Some knockout cells exhibit multi-nuclei, indicating problems in cell dividing and cell cycle progression. These observations suggest certain level of Rab43 is required for normal cell growth and cell cycle progression. To further reveal the molecular mechanism underlying Rab43 mediated regulation, the Akt activation in control (V2 vector transfected) and Crispr-Rab43 HepG2 cells was examined under normal culture conditions. We found the basal level of Akt activation was barely detectable in Crispr-Rab43 cells without any stimulation (Fig. 4e), rendering the reasonable comparison of Akt activation between the two cell lines unfeasible. Thus, we switched to look at the activation of Akt following insulin stimulation. Despite the fact that little activated Akt was detected in the insulin stimulated Rab43 knockout cells, Akt activation was dramatically increased in wild type Rab43 control cells following insulin stimulation, whereas the levels of total Akt protein in both cell lines were almost equal (Fig. 4e-g). Moreover, a similar pattern of the phosphorylation of p70S6K, a downstream target of Akt signaling, was observed (Fig. 4f-g), suggesting Akt-mTOR-p70S6K signaling is impaired upon loss of Rab43. Since it is well known that Rab43 involves in ER-Golgi trafficking, our data point to a potential role of protein trafficking in cell transformation and tumorigenesis.

### High levels of Rab43 protein are correlated with the familial cancer syndrome

**Fig. 5:**
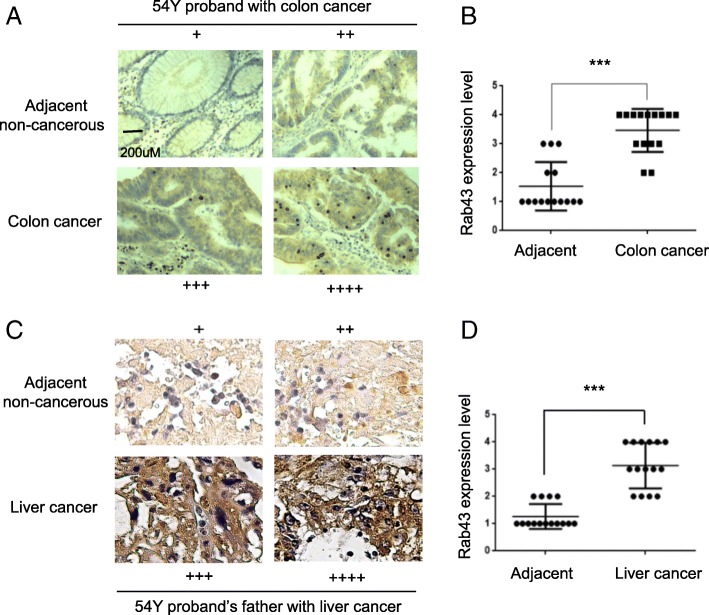
High levels of Rab43 protein expression were detected in the liver and colon cancer tissues, but not in their adjacent non-cancerous tissues (**a-b**) Representative Rab43 staining images in adjacent non-cancerous and colon cancer tissues obtained from the 54Y proband. **a** Rab43 staining score was displayed beside each image. The average levels of Rab43 protein from 15 adjacent non-cancerous colon tissue sections (slides) or 15 colon cancer tissue sections (slides) were summarized and compared in (**b**), ***p < 0.001 by Mann–Whitney test. **c-d** Representative Rab43 staining images in adjacent non-cancerous and liver cancer tissues from the proband’s father, the average levels of Rab43 protein from 15 adjacent non-cancerous liver tissue sections (slides) or 15 liver cancer tissue sections (slides) were summarized and compared in D, ***p < 0.001 by Mann–Whitney test

To further confirm the relevance of identified Rab43 variant to the onset of the studied familial cancer syndrome, we performed immunohistochemistry in colon tissues of the 54Y proband with colon cancer and in liver tissues of his farther died from liver cancer. We found high levels of Rab43 protein occurred in both colon and liver cancer tissues, whereas much less of Rab43 was detected in their adjacent non-cancerous tissues (Fig. 5a-c). Eventually, a total of 15 cancerous or non-cancerous tissue sections (slides) from the proband or his father were analyzed, the overall expression difference of Rab43 protein in between cancer tissues and non-cancerous tissues is statistically significant (Fig. 5b-d) (*p* < 0.001). These results clearly demonstrated there is a positive correlation between Rab43 protein expression level and the development of the familial liver- colon cancer syndrome. Given the fact that the proband and his father were heterozygous carriers of the Rab43 mutant allele, we are not sure whether the high levels of Rab43 observed in cancer tissues is contributed by elevated expression of Rab43 A158T alone. Nevertheless, the positive correlation

between Rab43 and cancer development in the family strongly hints Rab43 A158T mutant might be the driving force for the onset of that familial liver-colon cancer syndrome.

## Discussion

To identify susceptible genes for hereditary diseases, especially, hereditary cancer syndromes, two classes of approaches including functional screening and DNA genetic analysis are often exploited [17, 18]. Functional screening consists of gain-of-function screens (e.g. human cDNA expression screens) and loss-of-function screens (e.g. antisense application, RNA interference and Crispr-Cas9 gene editing) [19, 20]. As to DNA genetic analysis, whole genome sequencing, whole-exome sequencing and genome wide genetic linkage analyses are employed mostly [21–23]. A couple of advantages are offered by whole-exome sequencing. First of all, this approach is a well-developed technology targeting coding genes only, which reduces the workload of DNA sequencing and data analysis significantly. Secondly, whole-exome sequencing is able to identify 100% heterozygous mutations at the 30x sequencing data coverage [24]. Lastly, the whole-exome sequencing data is less complex and more reliable compared to that of whole genome sequencing. As such, we used whole-exome sequencing to search for potential susceptible gene candidates for that familial liver and colon cancer syndrome.

Despite the fact that a large amount of efforts have been put into the identification of causal genetic mutations susceptible to hereditary cancers, however, there are still many unknown rare high-penetrance mutations predisposing to these cancers to be discovered yet [25, 26]. The recently discovered germ-line mutations in POLE and POLD1 genes from individuals with multiple colorectal adenomas, carcinoma, or both are excellent examples in support of above assumption [27]. Several layers of evidence indicate the familial liver-colon cancer syndrome studied by us differs from canonical hereditary colorectal cancer (CRC) and Lynch syndrome. First, we did not find any germline mutations in both APC and MUTYH genes [4, 28], two major causal mutations for CRC. Secondly, we also failed to identify mutations from mismatch repair genes including MLH1, MSH2, MSH6 and PMS [29, 30], which are major susceptible genes for Lynch syndrome. Thirdly, our studied family was affected by two types of cancers. As of the date when blood samples were collected, four persons were diagnosed with liver cancer and two persons were diagnosed with colon cancer in that family. These two diseases spanned in three consecutive generations display an obvious autosomal dominant inheritance pattern. So far, no such diseases were discovered in the fourth generation of that family, most likely due to their young ages. Lastly, our functional analysis demonstrated the property of Rab43 variant is oncogenic, in sharp contrast to the loss-of-function nature of mutations occurred in APC, MUTYH and mismatch repair genes, etc.

How a germline mutation on Rab43 gene is susceptible to hereditary cancer remains unclearly currently. Rab43 is a member of small GTPase family including Ras oncogene. The known function of Rab43 is to facilitate the ER-Golgi transportation of newly synthesized proteins in coordination with Arf/Arl/Rab family members [11, 31]. Additionally, Rab43 has been implicated in the Shiga toxin trafficking from cellular membrane to Golgi and biogenesis of Golgi complex [13, 32]. We demonstrated Akt activation requires intact Rab43. Upon loss of Rab43, there is a little of activated Akt, whereas the total level of Akt protein remains unchanged (Fig. 4e-f), suggesting some of the Rab43 mediated functions might be required for normal activation of Akt. Furthermore, Rab43 A158T mutant would promote Rab43 functions and stimulate the activation of Akt, leading to enhanced cell proliferation and transformation. By contrast, loss of Rab43 protein would impair Rab43 involved processes and impede the Akt activation. In support of our working model of Rab43 regulating Akt activation, Akt has been reported to facilitate Rab5 mediated endocytosis [33] and is able to promote the phosphorylation of Rab43 in pancreatic duct carcinoma cells [34]. Towards this end, more vigorous investigations are in need in the future.

On the other hand, proteins involving in ER-Golgi trafficking have been implicated in tumorigenesis. High level expression of Rab43 was reported in Chinese glioma cancer patients and correlated with the disease progression and poor prognosis [16] and cell adhesion and cell migration are also affected by Rab43 and other small GTPases [35]. Moreover, the oncogenic nature of Rab43 A158T mutant demonstrated by our experiments is in perfect agreement with the autosomal dominant pattern of inheritance of this familial liver-colon cancer syndrome, indicating a driving role of the Rab43 mutation in the onset of that hereditary cancer syndrome.

## Conclusions

In summary, by using whole-exome sequencing, we identified that a germline mutation occurred on Rab43 gene is associated with the predisposition to a Chinese familial liver and colon cancer syndrome, exploring this mutant allele will not only help better understand the potential role of protein trafficking in tumorigenesis of this familial cancer syndrome, but also facilitate the genetic counseling to the affected families and offer more feasible prevention strategies in the clinic.

## Additional file

Additional file 1: **Figue S1.** Sanger sequencing confirmed the Rab43 knockout HepG2 cell line harbors three Rab43 mutant alleles with reading frame shift mutations in the gRNA targeting region. Sanger sequencing results are presented by ChromasPro software in the upper panel, a total of three Rab43 mutant alleles were identified as listed in the lower panel. Mutant-1 allele harbors a deletion of A, mutant-2 allele with a deletion of C and mutant-3 allele with an insertion of C before PAM consensus sequence in comparison with the sequence of wild type Rab43 allele. (PDF 75 kb)

## Data Availability

Data and materials are available upon request.

## Abbreviations

A158T: Alanine 158 Threonine
Crispr: Clustered regularly interspaced short palindromic repeats
ER: Endoplasmic reticulum
G12 V: Glycine 12 Valine
IHC: Immunohistochemistry
Indels: Insertions and deletions
MTT: 3-(4,5)-dimethylthiazol (− 2-y1)-2,5-di- phenyltetrazolium bromide
SNVs: Single nucleotide variants
