# Supplementary material for: A germline mutation in Rab43 gene identified from a cancer family predisposes to a hereditary liver-colon cancer syndrome
Source: BMC Cancer. 2019 Jun 21;19:613. doi: 10.1186/s12885-019-5845-4 (PMC6588942; doi:10.1186/s12885-019-5845-4)
Supplement: Supplementary file 1 — Figue S1. Sanger sequencing confirmed the Rab43 knockout HepG2 cell line harbors three Rab43 mutant alleles with reading frame shift mutations in the gRNA targeting region. Sanger sequencing results are presented by ChromasPro software in the upper panel, a total of three Rab43 mutant alleles were identified as listed in the lower panel. Mutant-1 allele harbors a deletion of A, mutant-2 allele with a deletion of C and mutant-3 allele with an insertion of C before PAM consensus sequence in comparison with the sequence of wild type Rab43 allele. (PDF 75 kb) [file 12885_2019_5845_MOESM1_ESM.pdf]

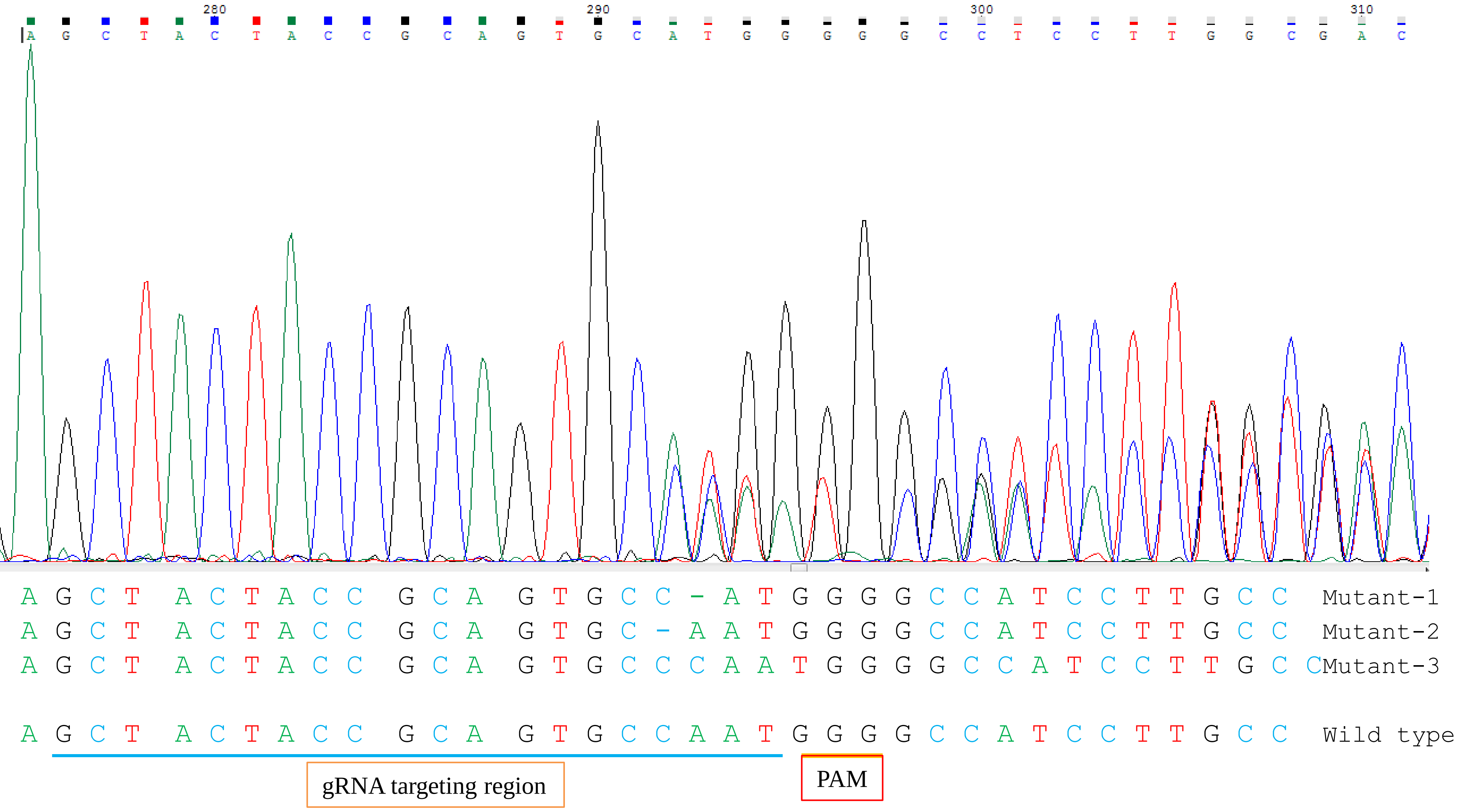

Fig. S1 Sanger sequencing confirmed the Rab43 knockout HepG2 cell line harbors three Rab43 mutant alleles with reading frame shift mutations in the gRNA targeting region

Sanger sequencing results are presented by ChromasPro software in the upper panel, a total of three Rab43 mutant alleles were identified as listed in the lower panel. Mutant-1 allele harbors a deletion of A, mutant-2 allele with a deletion of C and mutant-3 allele with an insertion of C before PAM consensus sequence in comparison with the sequence of wild type Rab43 allele.
